# Evaluation of the accuracy of two point-of-care haemoglobin meters used in sub-Saharan African population: a cross-sectional study

**DOI:** 10.1186/s12872-020-01371-x

**Published:** 2020-03-05

**Authors:** Simeon-Pierre Choukem, Colette Sih, Akondu Tontu Ntumsi, Christian Akem Dimala, Yannick Mboue-Djieka, Eveline D. T. Ngouadjeu, Andre-Pascal Kengne

**Affiliations:** 1 Department of Internal Medicine and Paediatrics, Faculty of Health Sciences, University of Buea, Buea, Cameroon; 2 Health and Human Development (2HD) Research Network, Douala, Cameroon; 3 Department of Internal Medicine, Douala General Hospital, P.O. Box 4856, Douala, Cameroon; 4 Faculty of Medicine and Pharmaceutical Sciences, University of Dschang, Dschang, Cameroon; 5 Infectious Disease Unit, University Hospitals of Leicester, Leicester, UK; 6 Haematology Unit, Department of Laboratory, Douala General Hospital, Douala, Cameroon; 7 Faculty of Medicine and Pharmaceutical Sciences, University of Douala, Douala, Cameroon; 8 South African Medical Research Council and University of Cape Town, Cape Town, South Africa

**Keywords:** Point-of-care Haemoglobin meter, Technical accuracy, Clinical accuracy, Agreement, Anaemia

## Abstract

**Background:**

Point-of-care haemoglobin meters are attractive solutions to improve timely diagnosis of anaemia in resource-limited settings. However, concerns regarding the accuracy of these meters may affect their adoption. The accuracy of two hand-held point-of-care haemoglobin meters was evaluated against reference full blood count analyser.

**Methods:**

This was a hospital-based cross-sectional study conducted at the Douala General hospital, Cameroon. Two handheld haemoglobin meters were assessed: Urit12® (URIT Medical Electronics Co.,Ltd. Guangxi, China) and MissionHb®(ACON Laboratories, Inc., San Diego, USA); against a reference standard CELL-DYN RUBY® (ABBOTT DIAGNOSTICS, Illinois, USA). The Pearson’s correlation and Bland-Altman agreement were used to assess the technical accuracy of the meters. Clinical accuracy was evaluated using total error allowable and area under the Receiver Operating Curve. Finally, their agreement with the reference in diagnosing anaemia was assessed using the kappa statistic.

**Results:**

A total of 228 participants were included in the study. The mean haemoglobin values of both haemoglobin meters (MissionHb®: 11.6 ± 2.5 g/dl; Urit12®: 10.9 ± 2.7 g/dl) were significantly higher than the reference value **(**10.5 ± 2.5 g/dl), *p* < 0.001 for both meters. Both haemoglobin meters had good correlation with the reference analyser (*r* = 0.89 and *r* = 0.90 for Urit12® and MissionHb® respectively) and good agreement on the Bland-Altman plots. However, the MissionHb® meter did not meet the clinical accuracy requirements (*p* < 0.001). Even though both meters were excellent at identifying the presence of anemia (MissionHb® - AUC = 0.9161, Urit 12® - AUC = 0.9009), they, however, both had weak agreement with the reference analyser in diagnosing the severity of anaemia (K = 0.39 for MissionHb®, *p* < 0.001 and K = 0.54 for Urit12®, *p* < 0.001).

**Conclusion:**

Although both devices showed technical accuracy with a positive correlation with the reference analyser and were able to accurately diagnose the presence of anemia, both meters however, had sub-optimal agreement with the reference analyser in diagnosing the degree of severity of anaemia among our participants.

## Background

Anaemia constitutes a major global public health problem. Globally, it currently affects two billion people (32.9% of the world’s population) [1]. However it is disproportionately concentrated in developing nations where it is four times more prevalent than in developed world [2]. Anaemia also results in one million deaths annually, with three-quarters of these occurring in Africa and South East Asia [3]. Anaemia is associated with major health consequences including decreased well-being, fatigue, lethargy, impaired physical activity and work performance [4]. In children it is associated with increased mortality and decreased cognitive and physical development [5, 6]. In pregnant women it is associated with low-birth weights, increased risk of maternal and perinatal mortality [7].

Despite the fact that the burden of anaemia is highest in developing nations, standard medical testing facilities are often limited and inaccessible to most patients [8]. Thus most cases of anaemia will go undiagnosed and untreated [9]. The economic realities in developing nations makes it cost-prohibitive to setup and run standard laboratory facilities at all levels of the health care system [8, 10, 11]. The use of point-of-care (POC) haemoglobin meters is potentially economically advantageous and time-saving and may also be of tremendous help in critical care units where rapid and frequent testing are required [11, 12]. These devices are much cheaper than automated analysers and do not require sophisticated expertise training to be able to operate them [13]. As such they are usually operated by a non-laboratory staff such as a physician who is directly involved in patient care [12]. They are battery-powered and will be highly beneficial in settings with inconsistent electric power supply. They are either bench-top or hand-held thus do not require sophisticated infrastructure [14]. They require smaller blood samples and thus are valuable in conditions that require multiple blood draws such as in critical care units and neonatal/paediatric units [12]. This reduces the risk of iatrogenic anaemia. They require capillary blood samples and will be appropriate in situations where it is technically difficult and distressing to perform phlebotomy [15].

Despite the potential benefits of using POC haemoglobin meters in clinical practice, there is still a lot of concern surrounding their accuracy [16, 17]. Do they underestimate haemoglobin values and thus expose patients to needless and sometimes harmful treatment for anaemia such as blood transfusions or do they overestimate haemoglobin values and as such prevent patients with anaemia from receiving appropriate treatment for anaemia? All these concerns have slowed their adoption in clinical practice. We aimed to evaluate the technical and clinical accuracy of two POC haemoglobin meters and their agreement with a reference analyser in diagnosing anaemia in the African setting.

## Methods

### Study design, setting and population

This was a hospital-based cross-sectional study carried out at the Douala General hospital over a period of 4 months from 1st November 2015 to 29th February 2016.

The Douala General hospital is a tertiary care hospital located in the city of Douala, the economic capital of Cameroon. Its laboratory is accredited by the World Health Organisation (WHO) and runs a 24-h shift every day. The laboratory is equipped with two automated full blood count (FBC) analysers: a Urit-3300®analyser (Urit medical Electronics Co. Ltd., Guangxi, China) and a CELL-DYN RUBY®analyser (ABBOTT DIAGNOSTICS, Illinois, USA). The laboratory participates in a three-monthly external quality control programme with peer laboratories in Douala and France.

All in- and out-patients, both children and adults, who were prescribed a full blood count during the study period were eligible to participate. This was irrespective of their underlying conditions, indications or the presence or absence of anemia. The etiology of anaemia in study participants, if present, was beyond the scope of this study. Coagulated blood samples and those who posed difficulty getting enough capillary samples to cover the test areas on the test strips were excluded from this study.

The study was approved by the Institutional Ethics Committee for Research on Human Health, University of Douala and administrative clearance obtained from the authorities of the Douala General Hospital.

### Selection of haemoglobin meters

The point-of-care haemoglobin meters were selected by contacting all hospital laboratories and distributors of health care products in order to ascertain all the brands are available in Cameroon. During this process, five meters were identified: MissionHb® (ACON Laboratories, Inc., San Diego, USA), Urit12® (URIT Medical Electronics Co.,Ltd. Guangxi, China), HemoCue® (HemoCue, Angelholm, Sweden), DHT® meter (Developing Health Technologies, Ipswich, UK) and Humameter® (HUMAN Medical Diagnostics, Wiesbaden, Germany). The last three meters are bench-top haemoglobin meters which function based on spectrophotometry with the use of microcuvettes and require expert operation. Hence, they were excluded from this study.

MissionHb® and Urit12® are both hand-held devices which use strip technology based on reflectance photometry and display haemoglobin values within 15 to 30 s of application of a drop of capillary blood.

### Data collection and Haemoglobin measurement

For each participant, data was collected on age, sex and pregnancy status. Then capillary haemoglobin was measured using standard operating procedures and in compliance with the manufacturers’ prescriptions. After letting the participant’s hand to hang vertically for 20 s to allow blood flow by gravity, the side of pulp of the middle finger is cleaned with an alcohol swab and then dried. It is pricked to obtain capillary blood and the first drop of blood wiped off. The respective strips were inserted into each meter and when indicated by the meter, a drop of blood was applied to the test spot. The meters then displayed the readings within 15 to 30 s. Measurements with the haemoglobin meters were done by a final year medical student. This was done concomitantly with collection of venous samples for full blood count analysis. A full ethylenediaminetetra-acetic acid (EDTA)-anticoagulated tube of venous sample was obtained from the median cubital vein by trained health personnel. The full blood count analysis was done using the CELL-DYN RUBY® according to its standard operating procedure, with daily quality control tests on both meters and the CELL-DYN RUBY®. The reference standard results were not available to the investigator all through the tests with the haemoglobin meters.

### Definition of terms

Anaemia was defined using the WHO age and sex-specific cut-off values of haemoglobin for mild, moderate and severe anaemia [18]. These cut-offs are shown on Table 1 underneath.

**Table 1:** Haemoglobin values for the diagnosis of anaemia

Population	No anaemia	Mild	Anaemia	Severe
			Moderate	
Children 6-59 months of age	110 or higher	100–109	70–99	Lower than 70
Children 4–11 years of age	115 or higher	110–114	80–109	Lower than 80
Children 12–14 years of age	120 or higher	110–119	80–109	Lower than 80
Non-pregnant woman (15 years of age and above)	120 or higher	110–119	80–109	Lower than 80
Pregnant women	110 or higher	100–109	70–99	Lower than 70
Men (15 years of age and above)	130 or higher	110–129	80–109	Lower than 80

### Units of haemoglobin in g/l

#### Statistical analysis

Data were analyzed using STATA 14 (Stata Corporation, Texas, USA). The various haemoglobin readings were summarized as means and standard deviations. Paired sample t-test was used to compare the mean haemoglobin value of each haemoglobin meter with the reference value. The continuous agreement between haemoglobin meters-based measurements and reference value was assessed with the use of Pearson’s correlation, and Bland-Altman plots. The Clinical Laboratory Improvement Amendments (CLIA) 2014 total error allowable desirable limits for haemoglobin measurement (± 4.19%) was used to assess the clinical accuracy of the haemoglobin meters [19]. Receiver Operating Curves (ROC) comparing both meters to the reference analyser in detecting the presence of anemia were plotted and the respective areas under the curve (AUC) and confidence intervals were calculated. Cohen’s kappa statistic was used to assess for the agreement of each meter with the reference analyser in diagnosing the severity of anaemia [20].

The study was reported according to the Standards for Reporting of Diagnostic Accuracy studies (STARD) guidelines, as per the STARD 2015 checklist [21].

## Results

A total of 228 participants (104 [45.6%] being men) with 8 (6.45% of women) being pregnant were included. Ages ranged from 4 to 85 years with a mean of 42.4 ± 17.3 years. The general characteristics of the study population are shown on Table 2.

**Table 2:** General characteristics of study participants recruited during this study

Characteristics	Overall	Women	Men
N(%)	228 (100%)	124 (54.4%)	104 (45.6%)
Age, years			
Mean (SD)	42.4 (17.3)	43.3 (17.0)	41.0 (17.6)
Median	40	39	40.5
25th–75th percentile	31–55	31.5–57.5	30.5–53.5
Min-Max	4–85	9–82	4–85
Age ranges, years			
4–11	08 (3.51%)	1 (0.44%)	7 (3.07%)
12–14	04 (1.75)	4 (1.75%)	4 (1.75%)
15–85	216 (94.74%)	119 (52.19%)	93 (40.79%)
Pregnancy	08 (3.51%)	08 (6.45%)	NA

Haemoglobin values obtained with the haemoglobin meters were significantly higher (*p* < 0.001) compared to the reference values (MissionHb® Mean ± SD [range]: 11.6 ± 2.5 g/dl, [4.5 to 24.6]; Urit12®: 10.9 ± 2.7 g/dl, [2.6 to 21.9]; reference analyser: 10.5 ± 2.5 g/dl, [2.9 to 20.7])

### Technical accuracy

**Fig. 1:**
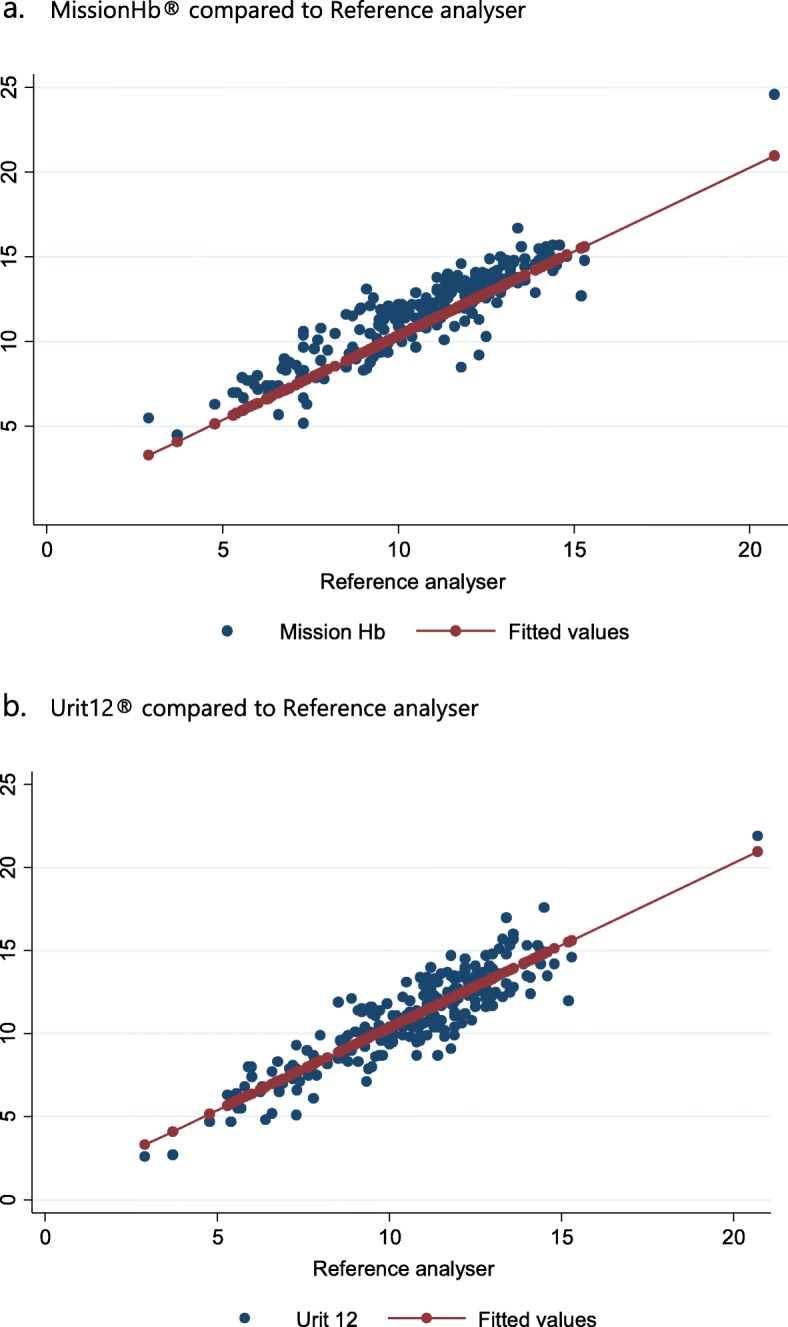
Scatter plot of MissionHb® versus reference haemoglobin values (Panel **a**) and Urit12® versus reference haemoglobin values (Panel **b**). X-axes: reference haemoglobin values (g/dl) Y-axes: MissionHb® or Urit12® haemoglobin values respectively

The differences in haemoglobin measurement between each haemoglobin meter and the reference analyser ranged from −3.3 to 4 g/dl with a positive mean bias of 1 g/dl for MissionHb® (*p* < 0.001) and − 3.2 to 3.6 g/dl with a positive mean bias of 0.3 g/dl for Urit12® (*p* < 0.001). A positive correlation was observed between the two POC meters and the reference analyser; with Pearson’s correlation coefficients 0.90 (MissionHb®) and 0.89(Urit12®). This is shown on Fig. 1. No significant difference between the two correlation coefficients was present (Steiger test: z = 0.46, *p* = 0.66).

**Fig. 2:**
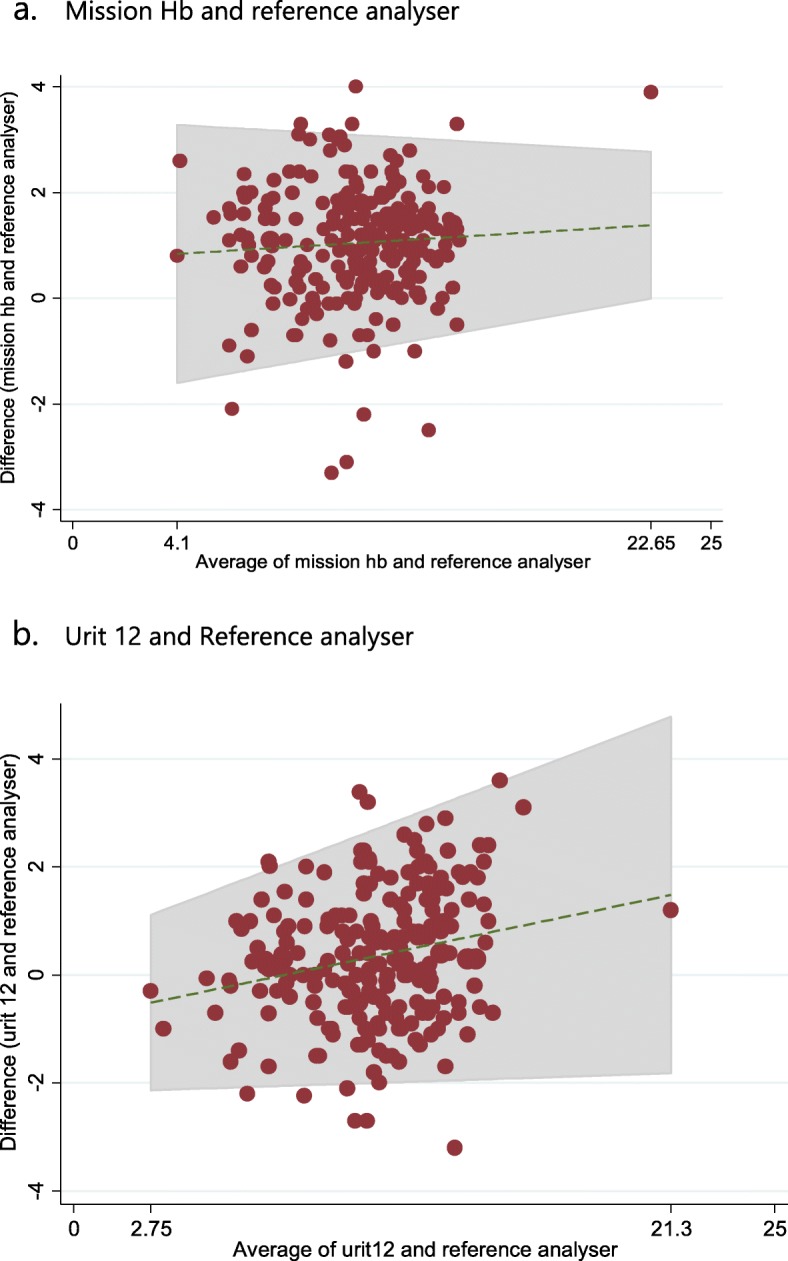
Bland-Altman graph of agreement between MissionHb® and the reference analyser (Panel **a**), Urit12® and reference analyser (Panel **b**). X-axes: average of respective POC and reference haemoglobin values (g/dl); Y-axes: differences in respective POC and reference haemoglobin values (g/dl). The middle horizontal lines represent the mean biases (g/dl) of the respective haemoglobin meters. The outer horizontal lines represent the respective 95% limits of agreement

Bland-Altman plots showing the agreement between the POC meters and the reference analyser are depicted in Fig. 2. MissionHb® meter had 7/228 (3.1%) of the difference scores out of the 95% limits of agreement (− 1.1, 3.2), with 2 values being in the positive region and 5 in the negative region. The mean bias was + 1 g/dl with a standard deviation of 1.1. The width of the 95% limits of agreement was 4.3 g/dl. Urit12® meter had 10/228 (4.4%) of the difference scores out of the 95% limits of agreement (− 2.1, 2.7), with 5 being in the positive region and 5 in the negative region. The mean difference (bias) difference was + 0.3 g/dl, with a standard deviation of 1.2. The width of the 95% limits of agreement was 4.8 g/dl.

### Clinical accuracy

Both haemoglobin meters overestimated haemoglobin values as their measured mean haemoglobin values were significantly higher than measured by the reference analyzer (Table 3). However, only the MissionHb® meter had mean haemoglobin values above the clinically allowable range when taking into consideration the desirable limit for bias in haemoglobin measurement (Table 3).

**Table 3:** Clinical Significance of the different Haemoglobin meter readings based on total allowable error

Haemoglobin meter	Subgroup	Measurement	Mean	SD	% difference	Statistical significance	TE%	Mean*TE%	Allowable range		Clinical significance
									Min	Max	
MissionHb®	Overall	Reference	10.5	2.5	0	< 0.001	4.19	0.4	10.1	10.9	Significant
		MissionHb	11.6	2.5	10.5						
	Women	Reference	10.2	2.1	0	< 0.001	4.19	0.4	9.8	10.6	Significant
		MissionHb	11.2	2.2	9.8						
	Men	Reference	10.9	2.8	0	< 0.001	4.19	0.5	10.4	11.4	Significant
		MissionHb	12	2.8	10						
Urit12®	Overall	Reference	10.5	2.5	0	< 0.001	4.19	0.4	10.1	10.9	Non-significant
		Urit12	10.9	2.7	3.8						
	Women	Reference	10.2	2.1	0	< 0.001	4.19	0.4	9.8	10.6	Non-significant
		Urit12	10.5	2.4	2.9						
	Men	Reference	10.9	2.8	0	< 0.001	4.19	0.5	10.4	11.4	Non-significant
		Urit12	11.4	3	4.6						

Mean (TE%) or total error allowable = Mean *TE%

### Agreement with the reference analyser in diagnosing the presence and severity of anemia

**Fig. 3:**
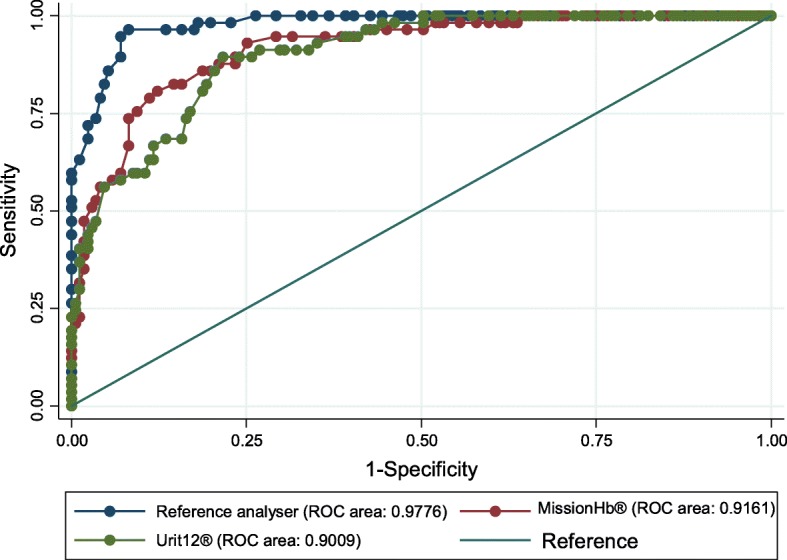
Receiver Operating Curve (ROC) comparing the MissionHb® and Urit 12® meters to the reference analyser

One hundred and seventy-one (75%) participants had anaemia with 62 (27.2%) being mild, 68 (29.8%) being moderate and 41 (17.9%) being severe, based on the results provided by the reference analyser. In general, both meters were excellent in diagnosing the presence of anemia as evidence by high AUC values for the ROC comparing them to the reference analyser (MissionHb® - AUC = 0.9161, Urit 12® - AUC = 0.9009) as summarised on Table 4 and Fig. 3. However, with regards to distinguishing between the degrees of severity of anaemia among the participants, both haemoglobin meters had weak agreement with the reference analyser (K = 0.39, *p* < 0.001 for MissionHb® and K = 0.54, *p* < 0.001 for Urit12®) as shown on Tables 5 and 6 below.

**Table 4:** Area Under the Curve (AUC) for the Receiver Operating Curves (ROC) comparing the MissionHb® and Urit 12® meters to the reference analyser

Meters	Area under curve (AUC) [95% CI]
Reference Analyser	0.9776 [09624–0.9929]
MissionHb®	0.9161 [0.8764–0.95586]
Urit 12®	0.9009 [0.8601–0.9416]

**Table 5:** Agreement in the diagnosis of anaemia using kappa statistics between MissionHb® and the reference analyser

Reference analyser	MissionHb®				
	No anaemia	Mild anaemia	Moderate anaemia	Severe anaemia	Total
No anaemia	**54**	2	1	0	57
Mild anaemia	38	**20**	4	0	62
Moderate anaemia	12	27	**29**	0	68
Severe anaemia	0	0	19	**22**	41
Total	104	49	53	22	**228**

Agreement: 54.82%; Expected Agreement: 25.92%; Kappa: 0.39; Standard Error: 0.0375; Z: 10.4; *P* < 0.001

**Table 6:** Agreement in the diagnosis of anaemia using kappa statistics between Urit12® and the reference analyser

Reference analyser	Urit12®				
	No anaemia	Mild anaemia	Moderate anaemia	Severe anaemia	Total
No anaemia	**48**	6	3	0	57
Mild anaemia	24	**25**	13	0	62
Moderate anaemia	6	15	**45**	2	68
Severe anaemia	0	0	8	**33**	41
Total	78	46	69	35	**228**

Agreement: 66.23%; Expected Agreement: 25.83%; Kappa: 0.54; Standard Error: 0.0384; Z: 14.19; *P* < 0.001

## Discussion

In our study we noticed a positive and significant correlation between each haemoglobin meter and the reference analyser (*r* = 0.90 for MissionHb® and *r* = 0.89 for Urit12®). However, both devices did not meet clinical accuracy requirements using total error allowable. Both meters significantly overestimated haemoglobin values and both had a weak agreement with the reference analyser in diagnosing the presence and severity of anaemia among our participants.

The positive correlation implies there was a strong direct linear relation between the measurements of each haemoglobin meter with those of the reference analyser. These results were comparable to that obtained for a bench-top haemoglobin meter-the HemoCue®(HemoCue, Angelholm, Sweden) by Lamhaut et al.*,* in France [17]. HemoCue® is the most popular and widely studied haemoglobin meter. Although other testing systems are available, scientific literature and on-field evaluation of non-HemoCue systems are extremely limited or even lacking for some 22]. Lamhaut et al.*,* included 44 participants undergoing potentially haemorrhagic surgery and they had a correlation of 0.85 between the HemoCue® and a reference analyser. The HemoCue® is technically more sophisticated than the devices and this similarity in results might mean that their simplicity does not compromise their technical accuracy [17]. Our results were lower than that obtained by Nkrumah et al.*,* in their study of the HemoCue® in Ghana [22]. They had a correlation coefficient of 0.995. This difference can be explained by the fact that the HemoCue® unlike the two devices has greater calibration functionality.

We also observed good agreement between the two haemoglobin meters and the reference analyser in the measurement of haemoglobin as shown on the Bland-Altman graphs. These results were better than those obtained by Schapkaitz et al.*,* in their study of the HemoCue® in South Africa [12]. Using a sample of 100 participants of all age groups, they had only 93% of the difference plots within the 95% limits of agreement (− 3.9, 5.5) and their limits of agreement were wider than those observed in our study. This difference can be explained by the fact that they used a smaller sample size than we did.

We also observed that the overestimated mean haemoglobin values, when compared to the reference analyser were statistically significant in both devices. However, only the MissionHb® meter had a mean haemoglobin significantly higher than the clinically allowable range. This implies that we could potentially fail to identify patients with borderline anaemia in some instances and provide adequate treatment with the use of these devices. Nevertheless, it is worth noting that both meters were overall excellent at identifying the presence of anaemia given their significantly high areas under the receiver operating curves. The ability of a haemoglobin meter to detect the presence or not of anaemia is very important in clinical settings, but its ability to determine the severity of the anaemia is even more important as the management of anaemia is entirely depending on its severity. Both meters had weak agreement with the reference analyser in determining the severity of the anaemia among our participants when we adopt a more logical interpretation of Cohen’s kappa statistic [20]. The respective levels of overestimation of haemoglobin by both meters might be an underlying reason for this finding. A weak level of agreement in diagnosing the severity of anaemia between both meters and the reference analyser, implies the severity of anaemia in several patients could be incorrectly assessed when using these meters and consequently lead to undue exposure of some patients to blood transfusions when not absolutely required. As such extreme clinical judgment would be imperative with the use of these devices.

Our study may be limited by a lack of a large representation of patients in the different ranges of anaemia (mild, moderate and severe). However, our study focused on hand-held haemoglobin meters thus adding to the extremely limited or even lacking literature of non-HemoCue systems. In addition, the results of our study may not be readily translated to all populations world-wide where the causes of anaemia may be very different from those of our study population (usually iron deficiency anemia). On the other hand, our study seems to be the first to assess the clinical implications of error in haemoglobin measurement using point-of-care haemoglobin meters.

## Conclusion

In conclusion, MissionHb® and Urit12® had good technical accuracy when compared to the reference analyser. However, the MissionHb® meter failed to meet clinical accuracy requirements, and both meters overestimated haemoglobin values. Even though both meters were excellent at detecting the presence of anaemia, they both had weak agreement with the reference analyser in determining the severity of the anaemia. Our findings suggest that although the use of these devices in resource-limited settings might offer enormous advantages compared to a reference analyser, extreme caution and clinical judgment are imperative to complement their use.

## Data Availability

The datasets generated during and/or analyzed during the current study are not publicly available (because some secondary manuscripts are still being written) but are available from the corresponding author on reasonable request.

## Abbreviations

CLIA: Clinical laboratory improvement amendment
FBC: Full blood count
POC: Point-of-care
WHO: World Health Organisation

## References

[1] United Nations System Standing Committee on Nutrition. Focusing on anemia. Available from www.unscn.org/web/archives_resources/files/Focusing_on_Anemia.pdf. Accessed 24 Feb 2020.

[2] Balarajan Y, Ramakrishnan U, Özaltin E, Shankar AH, Subramanian S (2011). Anaemia in low-income and middle-income countries. Lancet.

[3] Kayode OO, Adeolu OO. In: Silverberg D, editor. Anaemia in Developing Countries: Burden and Prospects of Prevention and Control: Anemia [Internet]. InTech; 2012. [cited 2015 Oct 15]. Available from: http://www.intechopen.com/books/anemia/anaemia-in-developing-countries-burden-and-prospects-of-prevention-and-control.

[4] WHO | Global Nutrition Targets 2025, editor. Anaemia policy brief [Internet]: WHO; 2015. [cited 2015 Oct 21]. Available from: http://www.who.int/nutrition/publications/globaltargets2025_policybrief_anaemia/en/.

[5] Brabin BJ, Premji Z, Verhoeff F (2001). An analysis of Anemia and child mortality. J Nutr.

[6] McCann Joyce C, Ames Bruce N (2007). An overview of evidence for a causal relation between iron deficiency during development and deficits in cognitive or behavioral function. The American Journal of Clinical Nutrition.

[7] Allen LH (2000). Anemia and iron deficiency: effects on pregnancy outcome. Am J Clin Nutr.

[8] Sharma S, Zapatero-Rodríguez J, Estrela P, O’Kennedy R (2015). Point-of-care Diagnostics in low resource settings: present status and future role of microfluidics. Biosensors.

[9] Brown J, Theis L, Kerr L, Zakhidova N, O’Connor K, Uthman M (2011). A hand-powered, portable, low-cost centrifuge for diagnosing Anemia in low-resource settings. Am J Trop Med Hyg.

[10] Drain PK, Hyle EP, Noubary F, Freedberg KA, Wilson D, Bishai W (2014). Evaluating diagnostic point-of-care tests in resource-limited settings. Lancet Infect Dis.

[11] St John A, Price CP (2013). Economic evidence and point-of-care testing. Clin Biochem Rev.

[12] Schapkaitz E, Mahlangu J (2011). Point-of-care estimation of haemoglobin concentration in all age groups in clinical practice. South African Family Practice.

[13] Briggs C, Kimber S, Green L (2012). Where are we at with point- of- care testing in haematology?. Br J Haematol.

[14] Price CP (2001). Point of care testing. BMJ.

[15] Sanchis-Gomar F, Cortell-Ballester J, Pareja-Galeano H, Banfi G, Lippi G (2013). Hemoglobin point-of-care testing the HemoCue system. J Lab Autom.

[16] Larsson A, Greig-Pylypczuk R, Huisman A (2015). The state of point-of-care testing: a european perspective. Ups J Med Sci.

[17] Lamhaut L, Apriotesei R, Combes X, Lejay M, Carli P, Vivien B (2011). Comparison of the accuracy of noninvasive hemoglobin monitoring by spectrophotometry (SpHb) and HemoCue® with automated laboratory hemoglobin measurement. J Am Dent Soc Anesthesiol.

[18] WHO. Haemoglobin concentrations for the diagnosis of anaemia and assessment of severity. Vitamin and Mineral Nutrition Information System. Geneva: World Health Organization; 2011. http://www.who.int/vmnis/indicators/haemoglobin.pdf. Accessed 14 Oct 2015.

[19] Westgard J. Desirable Biological Variation Database specifications - Westgard. https://www.westgard.com/biodatabase1.htm. Accessed 16 Oct 2017.

[20] McHugh ML (2012). Interrater reliability: the kappa statistic. Biochem Med.

[21] Equator Network. Standard for Reporting of Diagnostic Accuracy Studies, STARD 2015: An Updated List of Essential Items for Reporting Diagnostic Accuracy Studies. https://www.equator-network.org/reporting-guidelines/stard/. Accessed 12 Oct 2019.

[22] Nkrumah B, Nguah SB, Sarpong N, Dekker D, Idriss A, May J (2011). Hemoglobin estimation by the HemoCue® portable hemoglobin photometer in a resource poor setting. BMC Clin Pathol.

